# Supplementary material for: Perspectives of people living with HIV on barriers to timely ART initiation following referral for antiretroviral therapy: A qualitative study at an urban HIV clinic in Kampala, Uganda
Source: PLOS Glob Public Health. 2023 Jul 26;3(7):e0001483. doi: 10.1371/journal.pgph.0001483 (PMC10370709; doi:10.1371/journal.pgph.0001483)
Supplement: S2 File — (PDF) [file pgph.0001483.s002.pdf]

Codebook

Themes	Sub-themes	Codes	Condensed meaning	Frequency(N=17)
Drug related barriers	Anticipated side effects and duration of ART medicines	Barr_drug_bigPill_size	Clients had fears for big pills	10
		Barr_drug_havingtotakeIntime	Clients had fears for taking drugs in time	11
		Barr_drug_ManyPillBurd	Clients had fears for many pills	10
		Barr_drug_myths_run mad	Clients had ARVs myths and misperceptions	2
		Barr_drug_SideEff	Clients had fears for pills side effects	12
Individual related barriers	Immediate denial with late acceptance of HIV positive results	Barr_Ind_badEmotions_distress	Clients experienced bad emotions and distress	10
		Barr_Ind_Fear	Clients experiencing fear	15
		Barr_Ind_Rej_denialRes	Clients rejected/denied results	6
		Barr_Ind_UseHerbs	Clients used herbs as remedy	2
		Barr_Ind_UsePrayer	Clients used prayer as remedy	4
	Stigma resulting in non-disclosure and discrimination	Barr_fear_Stigma_discrimination	Clients feared stigma and discrimination from the community	13
		Barr_Ind_noFriends_Supp	Clients had no friends' support	6
		Barr_Ind_noPartner_Supp	Clients had no partners' support	4
Health system related barriers	Long waiting time and procedures at the ART clinic	Barr_Syst_Initial_Long procedure	Clients experienced length initial procedures at HIV Clinics	5
		Barr_Syst_LongWaitingTime	Clients experienced long waiting time	3
		Barr_Syst_manyClients	Health facility experienced high volume of clientele	4
	Negative experiences at the site of testing HIV positive	Barr_Syst_noCounsel_PrivClinic_work	Clients experienced poor counselling at the private clinics	3
		Barr_Syst_poorReferral	Clients experienced poor referral	7
