# Supplementary material for: Perspectives of people living with HIV on barriers to timely ART initiation following referral for antiretroviral therapy: A qualitative study at an urban HIV clinic in Kampala, Uganda
Source: PLOS Glob Public Health. 2023 Jul 26;3(7):e0001483. doi: 10.1371/journal.pgph.0001483 (PMC10370709; doi:10.1371/journal.pgph.0001483)
Supplement: Attachment — Submitted filename: Response to Reviewers.pdf [file pgph.0001483.s003.pdf]

Dr. Micheal Kiyingi

Makerere University College of Health Sciences

P. O Box 7062,

Kampala – Uganda

The Academic Editor

PLOS Global Public Health.

Date: March 22, 2023

Dear Sir/Madam,

RE: **Response to reviewer comments about our manuscript titled**, "*Perspectives of people living with HIV on barriers to timely ART initiation following referral for antiretroviral therapy: A qualitative study at an urban HIV clinic in Kampala - Uganda".*

On behalf of the authors, I write to thank you and the reviewers for providing these insightful comments to make this manuscript better and fit for publication in your journal. In this document, kindly find the responses to reviewer comments in the section below.

Yours Sincerely,

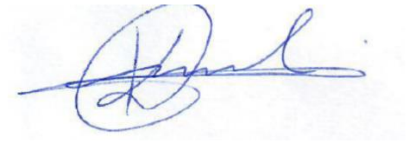

Dr. Micheal Kiyingi; MBChB, MMED.

Tel: +256777164339

Email: michealkiyingi@gmail.com

Reviewer #1: Thank you for the opportunity to review this manuscript. Delayed ART initiation and non-adherence to HIV treatment are major public health issues, particularly in sub-Saharan Africa. While the study addressed a significant problem in HIV care engagement, the manuscript however, needs major revisions in the methods, results, and discussion sections for it to be publishable. I have detailed some concerns that the authors need to address below.

Abstract

Line 41: Add information about the location of the study.

Response: Thank you for this comment. The study was conducted at Mulago ISS clinic in Uganda, and has been added.

Line 42: Specify the age range of study participants in parenthesis after "adult patients"

Response: Thank you. The age of ≥18years has been added.

Background

Line 63: Change "A" in "Anti-retroviral therapy" to a small letter "a"

Response: Thank you. This has been changed as suggested.

Line 63: Change "P and L" in "People Living" to a small letters.

Response: These have been edited to small letters.

Line 76: What does "flexible" HIV testing mean? Either hash out what it means or simply remove it from the sentence.

Response: 'Flexible' has been removed.

Line 78: Change "an HIV" to "a HIV"

Response: This has been changed as suggested.

Line 84/85: Revise phrasing "some patients may delay to present to the HIV clinic" for clarity. Please specify if you mean patients delay going to the clinic to initiate ART or if they delay clinic visits in general.

Response: Thank you for this comment. This has been clarified to 'delay to visit the HIV clinic to initiate ART'.

Methods

Line 104: I understand the meaning in context of "presented to Mulago" but I suggest that you revise this to "visited Mulago", which is a terminology that is commonly used in HIV/health research.

Response: Thank you for this comment. The section has been updated to 'visited'.

Data collection
Line 112-119: This section needs improvement, the description of data collection procedures is unclear. The authors mentioned in the previous section that purposive sampling was used to recruit participants into the study, however did not explain/describe how. Please clarify where interviews were conducted (e.g. at clinic, community, etc) and by whom?

Response: Thank you for this comment. The purposive sampling section has been updated. In addition to eligibility, participants were selected based on age group [18 – 24 (5 participants), 25 – 40 (9 participants), >40 (3participants)], sex (8males and 9 females) and place of HIV diagnosis (public health facility – 5, private health facility – 9, community outreach - 3). The interviews were conducted at the clinic in the counselling rooms by two social scientists. This section has been updated in the manuscript.

Results
Line 141/142: Please provide a table showing socio-demographic characteristics of study participants. This information is very important for context and helps other researchers/readers to better understand the population of interest and interpretation of study findings.
In addition, since findings in both the abstract and data analysis sections are being presented and interpreted according to the socio-ecological model (SEM). For consistency, authors need to revise/reorganize the themes in the results section to reflect the SEM framework i.e. individual level, interpersonal, organizational, etc.

Response: Thank you for this comment. The socio-demographic characteristics table has been added. We have re-organized our results according to the socio-ecological model at 3 levels, individual, interpersonal and organizational.

Line 177: Revise "report to the ART clinic" and use clearer phrasing e.g. "seek treatment/medical care".

Response: This has been updated to 'seek treatment at the HIV clinic'

Line 183-186: add quotation marks to participant's response.
Response: Thank you for this. The quotation marks have been added.

Line 202: Revise descriptors for consistency. Pick one format (Female, 35 years) or (Female PLHIV, 23 years) and revise all the other descriptors throughout this section.

Response: Thanks for pointing this out. Since all our participants were known HIV patients attending an HIV clinic, we decided to remove the PLHIV in all descriptors in this section.

Discussion
Line 242: It is unclear which themes have been identified at the interpersonal level. Here

authors' discuss findings "at individual and interpersonal levels" yet in previous sections, all 5 themes were classified as either "at the individual level or organizational level". Please revise the results section and clarify which themes were identified at the individual, interpersonal, and organizational levels.
Response: The results section has been updated to fit the SEM model and themes re-organized to individual, interpersonal and organizational levels.

Line 250: Revise sentence and change "he/she is" to "they are"
Response: This statement has been revised to avoid use of 'he/she'.

Line 253: Revise "individual and interpersonal levels", see comment above.
Response: These two sections have been discussed together because most of our findings at this level are similar to those in previous studies and most have findings falling in both these theme categories. However, we described the challenges at organizational level first, because most of the barriers we found at individual and interpersonal levels were similar to those found by previous research.

Line 260: Revise "At intrapersonal and interpersonal levels"
Response: This section has been revised.

Line 267: Provide a reference (s) to support this statement "Important to note, for the population we studied, the risk of loss to follow up after testing HIV positive is quite high…"
Response: Thank you for this comment. To our knowledge, there are no studies to answer this question in our setting or similar settings. The statement was based on operational observation rather than research. We have revised this section and removed the statement.

Conclusions
Line 290-291: This conclusion is not entirely supported by the data presented. Please see comment above regarding categorization of themes using the SEM.

Response: The results section has been revised and themes re-arranged as per socio-ecological model.

Reviewer #2:

I found major technical issues pertaining to various sections of this manuscript:
1. sampling vs. population were not clearly explained. How did the study reached the saturated number of informants?
Response: Thank you for the review. Sampling was purposive where we varied our population based on sex, age group and place of HIV diagnosis (i.e public health facility, private health facility or community outreach). As the interviews proceeded, preliminary data review and analysis was conducted and saturation was determined when no new information was obtained and we could derive categories from the data we have. This section has been revised for clarity in the newly uploaded manuscript version.

2. Instrument: the semi-structured questions were developed or adopted from which study / literature?
Response: The semi-structured questions were developed based on prior studies on barriers to ART conducted in East Africa and similar settings. The references are numbered 14 – 16 and 22 – 23.

3. Need to clearly describe the back translation process as this involve more than one language. This step is crucial to ensure the authenticity of the original expressed language with the intended meaning conveyed during the translation.
Response: Thank you for pointing this out. The back translation was done by the research assistants who conducted the interviews. This process has been described in the methods section.

4. What is the saturation point for the informants?
Response: The saturation point was reached when study participants resounded the same information as described in the response in the first comment of this section.

5. The main literature use to frame this study was an old study conducted in 1988. Whereby in today's society, social media disrupt and create a whole set of challenges to health communication. The study did not mention this and provide satisfactory comparative analysis to validate their reason/s of using an old study as point of reference.
Response: Thank you for this comment. The study in question is in reference to the article describing the socio-ecological model in health and behavior by McLeroy and colleagues. To our knowledge, this model and how it was proposed in that article has not been significantly modified to date, and many recent studies using this model reference it. We agree with the new trends in technology that can influence behavior and health in today’s society, but to our knowledge, this has not been studied, documented and incorporated in the model.

6. When analysis was conducted using Atlas ti, surely the presentation of result would have fineness in terms of table, abstraction processes, table of frequency, etc.
Response: The frequency table has been added to the manuscript. The abstraction process as aided by Atlas.ti has been clarified in the methods section (138 - 141). A table of frequencies of the codes and their respective themes and sub-themes has been provided as an attachment file (SF 3). The developed codes were grounded in data and revisions in response to emerging patterns were made.

7. The results were too shallow and did not succinctly provide a manifest and latent analysis of the prescribed themes.
Response: Thank you for this comment. The population we studied is relatively small and a prior quantitative study we conducted showed that these were only 15% in a large HIV clinic (Kiyingi et al. 2023). However, since we are moving to initiate everyone on anti-retroviral therapy and higher HIV treatment targets needed by 2030 (end AIDS), it was important to qualitatively study this population. The results section has been re-arranged to fit the socio-ecological model levels of influence which gives better depth.

8. Introduction to results was too simple.
Response: An introduction to results has been revised and a frequency table added after highlighting the key characteristics of participants.

9. If the extraction of quotations / analysis were done using Atlas ti, the source would be identified in more detail manners with the inclusion of percentile, minutes of the quotation, and discourse unit.
Response: A table of themes, sub-themes, codes and their frequencies has been added as an attachment file (SF 3). Discourse analysis was not followed for this study but we rather followed thematic/framework analysis techniques.

10. Out of 38 references, only 21 were latest five years studies. The rest were old. Recommend for revise and add latest citations.
Response: Thank you for this comment. The study focused on barriers to timely ART initiation using the test and treat (TAT) strategy as reference. Four of the references were evidence for TAT (Cohen 2011, Baeten 2012, Grinsztejn 2014 and Samji 2013). The TAT strategy was rolled out in 2015 by WHO and adopted in Uganda in 2016 (which are all more than five years ago). All 19 references on barriers to ART were after 2016 (16 are 2018 and above) which is seven years ago when test and treat was implemented and most were within five years. Only 3 old references that describe the methodology used to analyze data have been cited and these have not been significantly modified ever since.

11. Some citations did not follow the required citation style. Revise.
Response: This has been revised. All citations have been updated to use the Vancouver style of referencing.

12. This manuscript should be sent for proofread for cohesion and clarity.

Response: Thank you for this comment. The manuscript has undergone proofreading using a free software.
